# Validation of an algorithm for left ventricular segmentation in 150 patients shows potential for further development towards fully automatic segmentation

**DOI:** 10.1186/1532-429X-15-S1-E30

**Published:** 2013-01-30

**Authors:** Jane Tufvesson, Joey F Ubachs, Katarina Steding-Ehrenborg, Marcus Carlsson, Hakan Arheden, Einar Heiberg

**Affiliations:** 1 Clinical Physiology, Skane University Hospital, Lund University, Lund, Sweden; 2 Numerical Analysis, Lund University, Lund, Sweden

## Background

Automatic segmentation of the left ventricle (LV) is desirable to assess the cardiac parameters end-diastolic volume (EDV), end-systolic volume (ESV), ejection fraction (EF) and left ventricular mass (LVM) since manual segmentation is time consuming and observer dependent.

A physiologically correct segmentation of the left ventricle requires careful consideration of the long axis displacement and the LV outflow tract which makes the myocardium non-circumferential in the basal slices. To detect the long axis displacement a constraint could be used to keep the LVM fairly constant over the cardiac cycle. However, in order to use this constraint, the error of the segmentation has to be low regarding both endocardial and epicardial borders in the non-basal part of the LV. Therefore, the purpose of this study was to improve and validate an automatic algorithm for LV segmentation in the non-basal part of the LV, as a first step towards fully automatic segmentation.

## Methods

Manual delineation was performed in all subjects and used as the reference method. An existing LV segmentation algorithm, implemented as a 3D+T deformable model, was modified by adding anatomical information, optimizing parameters and adding a constraint to keep the papillary volume constant over time. A training set of short-axis SSFP image stacks from 50 subjects (n=27 patients with known or suspected coronary artery disease, n=15 healthy volunteers, n=8 athletes) was used in the optimization and the error in EDV, ESV and LVM to manual delineation was minimized. For validation of the algorithm the automatic segmentation was used in all slices except the most basal slice and basal slices with a non-circumferential myocardium, in which the manual delineation was used instead. The algorithm was validated by comparison of the difference in EDV, ESV, EF and LVM expressed as percentage (mean ± SD) in a test set of 150 subjects (n=81 patients, n=45 healthy volunteers, n=24 athletes).

## Results

**Figure 1:**
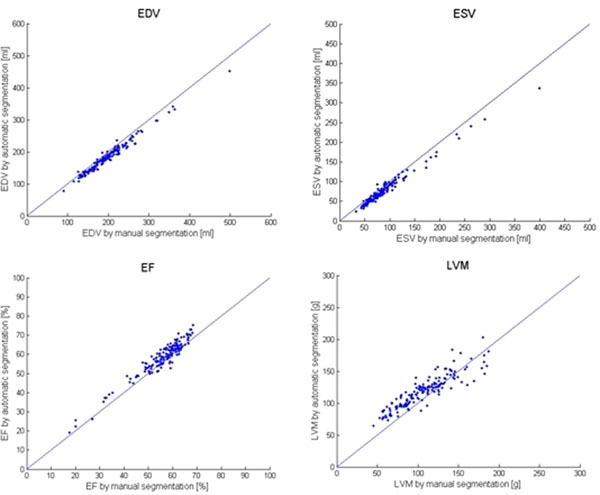
Automatic segmentation plotted against manual delineation for end-diastolic volume (EDV, top left), end-systolic volume (ESV, top right), ejection fraction (EF, bottom left) and left ventricular mass (LVM, bottom right).

The difference between automatic segmentation and manual delineation was -7.3 ± 4.1% (EDV), -12.4 ± 8.3% (ESV), 4.8 ± 5.5% (EF), and 14.2 ± 16.0% (LVM) (Table 1). The correlation between automatic segmentation and manual delineation was R=0.99 (EDV), R=0.99 (ESV), R=0.96 (EF), and R=0.90(LVM) (Figure 1).

**Table 1:** Results expressed as error between manual delineation and automatic segmentation in percentage and absolute error and as regression R-value for end diastolic volume (EDV), end systolic volume (ESV), ejection fraction (EF) and left ventricular mass (LVM).

	Error (mean±SD)	Absolute error (mean±SD)	Regression R-value
EDV	-7.3 ± 4.1 %	-14.4 ± 9.0 ml	0.99
ESV	-12.4 ± 8.3 %	-10.8 ± 8.7 ml	0.99
EF	4.8 ± 5.5 %	2.5 ± 2.7 %	0.96
LVM	14.2 ±16.0 %	11.3 ± 14.4 g	0.90

## Conclusions

The presented algorithm for segmentation of the non-basal part of the left ventricle shows a good agreement with manual delineation and a low to fair bias for EDV, ESV, EF and LVM. The algorithm has potential for further development to segmentation of the whole ventricle including the basal part.

## Funding

Swedish Research Council (2008-2949)

